# Analysis of nicotine, tar, carbon monoxide, total particulate matter, water, benzo[a]pyrene, and humectants in cigarettes and bidis from India and Myanmar

**DOI:** 10.1038/s41598-026-35417-5

**Published:** 2026-01-30

**Authors:** Priyamvada Sharma, Jagdish Kaur, Arvind Vashishta Rinkoo, Vijayashree Rao, Amina Salam, Ranti Fayokun, Pratima Murthy

**Affiliations:** 1 Department of Clinical Psychopharmacology and Neurotoxicology, National Institute of Mental Health and Neurosciences (NIMHANS), Bangalore, India; 2 Department of Healthier Populations and Noncommunicable Diseases, WHO Regional Office for South-East Asia, 6th Floor, Red Fort Capital Parsvnath Tower-1, Bhai Vir Singh Marg, Gole Market Sector-4, New Delhi, India; 3 Toxicology Laboratory, Centre for Addiction Medicine, Department of Psychiatry, National Institute of Mental Health and Neurosciences (NIMHANS), Bangalore, India; 4 Health Promotion Department, World Health Organization (WHO), Geneva, Switzerland; 5 National Institute of Mental Health and Neurosciences (NIMHANS), Bangalore, 560029 India

**Keywords:** Nicotine, Tar carbon monoxide, Total particulate matter, Benzo[a]pyrene, Water, Humectants, Flavours, Cigarettes, Bidis, Health care, Chemistry

## Abstract

Tobacco use poses a major public health challenge in the World Health Organization’s South-East Asia Region, where it contributes to approximately 2.3 million deaths each year. In 2020 alone, tobacco smoking was responsible for around 1.6 million of these deaths. The region faces a dual burden of high prevalence of both smoking and smokeless tobacco use, underscoring the urgent need for strengthened tobacco control measures. The toxic substances found in the emissions of smoked tobacco products are inadequately researched. This study presents primary scientific information on levels of nicotine, water, and benzo[a]pyrene (BaP) in mainstream smoke deliveries from popular cigarettes from India and Myanmar, and bidis from India; additionally, flavours and humectants were tested in fillers. Globally accepted methods from the World Health Organization’s Tobacco Laboratory Network (TobLabNet), the Centers for Disease Control and Prevention (CDC), and the Cooperation Centre for Scientific Research Relative to Tobacco (CORESTA) were used. When comparing Indian and Myanmar cigarettes, we discovered that nicotine and carbon monoxide (CO) levels in Myanmar cigarettes were slightly higher than those in Indian ones, though the difference was not statistically significant. Water, tar, and total particulate matter (TPM) also exhibited no statistically significant variations. Significantly higher *(p* = *0.008)* concentrations of BaP, ranging from 8.02 to 14.90 ng/cigarette (median, 9.95 ng/cigarette), were observed in Myanmar-origin cigarettes, indicating increased exposure risks for users. Among humectants, only propylene glycol showed significant variation (*p* = 0.023). Compared with Indian cigarettes, bidis showed significantly higher nicotine and CO (*p* = 0.023), as well as water and TPM (*p* = 0.008). When bidis were compared with cigarettes from both countries, nicotine (p = 0.041), water, and TPM differed significantly (both *p* < 0.001). The intended flavours were not detected in the mainstream smoke of the cigarettes and bidis examined. The findings of this study can be leveraged to enhance public health by identifying harmful chemicals that exceed established limits and potentially motivating manufacturers to produce less harmful products by conforming to toxicant emission standards.

**Supplementary Information:**

The online version contains supplementary material available at 10.1038/s41598-026-35417-5.

## Introduction

Tobacco use, including smoking, causes over 8 million deaths each year, with over 7 million from direct tobacco use, 1.6 million from smoking, and 1.2 million from passive smoking^1^. On average, smoking shortens life expectancy by about 10 years^2^. Tobacco use is a major threat to public health; it kills around 2.3 million people every year in the World Health Organization (WHO) South-East Asia Region (SEAR)^1^. As per the fifth edition of the WHO global report on trends in the prevalence of tobacco use 2000–2030^3,4^, the WHO SEAR is home to around 199 million tobacco smokers (20% of the global total) and 280 million smokeless tobacco users (77% of the global total). Tobacco is consumed in diverse forms, including smoked products such as cigarettes and bidis (a cheaper hand-rolled form of smoked tobacco), and a variety of smokeless tobacco. Comprehensive awareness of tobacco’s harmful effects is far from desirable among users due to culture-related customs and misbeliefs about their health effects^3^.

All forms of tobacco are harmful, and there is no safe level of exposure to tobacco. Cigarette smoking is the most common form of tobacco use worldwide. Other smoked tobacco products include waterpipe, cigars, cigarillos, heated tobacco products, roll-your-own tobacco, pipe tobacco, bidis, and kreteks. Around 80% of the 1.3 billion tobacco users worldwide live in low and middle-income countries^4^, where the burden of tobacco-related illness and death is heaviest. Tobacco use contributes to poverty by diverting household spending from basic needs such as food, education, and shelter to buying tobacco products^5^.

India is the second-largest consumer and producer of tobacco in the world. The Global Adult Tobacco Survey 2017 revealed that 28.6% (around 266 million) of adults in India, aged 15 and above, currently use tobacco in some form. Of these, 10.7% (around 99 million) currently smoke tobacco and 21.4% (around 199 million) use smokeless tobacco^1,2^. Tobacco products increase the risk of heart and lung diseases, as well as cancers and a variety of other adverse health outcomes^4^. India’s GDP is negatively impacted by tobacco use, and the direct medical expenses incurred in treating tobacco-related illnesses account for 5.3% of the nation’s yearly spending on both public and private health care^5^. It is estimated that the economic costs of tobacco-related diseases are ten times higher than the amount of money India receives from tobacco taxes^5^. This drains the public health system and the economy, which no country can afford. In the SEAR, Myanmar reports the highest tobacco use, with over half of adults (54%) using some form of tobacco. STEPS 2014 survey further revealed that 26% of adults in Myanmar were tobacco smokers and 43% used smokeless tobacco^2,4^. India and Myanmar can avert millions of preventable deaths as well as mitigate the damage of tobacco use on both society and the economy through the implementation of a comprehensive package of strong tobacco control policies outlined in the WHO Framework Convention on Tobacco Control (FCTC)^5^.

Nicotine in tobacco is responsible for its addictive nature^1^. Nicotine is a paradoxical substance because it has both stimulating and depressant effects. It affects the cardiovascular, skeletal, gastrointestinal, and peripheral nervous systems, among other systems^5^. At the molecular level, nicotine in tobacco mimics the neurotransmitter acetylcholine. Some of the effects of nicotine may be traced to its many receptors in cholinergic binding sites in the brain. Nicotine could be the main psychoactive component of tobacco due to its intricate pharmacological actions^6^. Several additional essences are purposefully added to tobacco products to make them attractive and to lessen their harshness and potential for dependence^2^. Many non-tobacco substances such as humectants, fragrances, and flavours are added to attract and appeal to consumers. The American Lung Association has released data indicating that there are roughly 600 ingredients in one cigarette. Almost 7000 chemicals are released when cigarettes burn, many of which are toxic. At least 69 of them are known to cause cancer^7^.

To ensure compliance with the WHO FCTC Articles 9 and 10, the contents and emissions of tobacco products must be monitored. The WHO Tobacco Free Initiative (TFI) established the tobacco testing laboratory network (TobLabNet) in 2005 as part of its global agenda. This is based on the recommendation of the WHO Study Group on Tobacco Product Regulation (TobReg)^8^ to build and strengthen tobacco product testing and research capacity by pursuing Articles 9 and 10 of the WHO FCTC^9^. Under WHO TFI initiative, TobLabNet develops and validates methods and standard operating procedures (SOPs) for testing the contents and emissions of tobacco products.

The regulation of maximum emission levels of tobacco product smoke, specifically tar, nicotine, and carbon monoxide (TNCO), is currently in progress in India. The Food Safety and Standards Authority of India (FSSAI) and the Central Drugs Standard Control Organization (CDSCO) have not yet formally mandated maximum TNCO limits in cigarettes or bidis. However, some tobacco manufacturers in India voluntarily adopt international standards such as 10 mg tar/1 mg nicotine/10 mg carbon monoxide (CO) per cigarette, following the European Union Directive 2001/37/EC^10^. A wide variety of smoked tobacco products are available in India and Myanmar; however, very little data on the emissions and addictiveness of smoked tobacco products are available from the region due to the infrastructure and technical expertise required for emission testing. This research aims to present primary scientific information on the amounts of nicotine, water, and benzo[a]pyrene (BaP) in tobacco emissions. Additionally, flavours and humectants were tested in fillers. Standardized and validated methods were employed.

## Material and methods

This study was carried out in India from June 2023 to May 2024 at the Tobacco Testing and Drug Toxicology Laboratory, Centre for Addiction Medicine (CAM), National Institute of Mental Health and Neurosciences (NIMHANS), Bangalore Karnataka. This tobacco testing facility is part of the WHO global network of tobacco testing laboratories.

All the chemicals used for analysis were of analytical grade. Nicotine, BaP, humectants (glycerol, propylene glycol, and triethylene glycol), flavours (methyl salicylate, ethyl salicylate, eugenol, diphenyl ether, cinnamaldehyde, menthol, coumarin, and camphor), internal standards (n-heptadecane, 3′,4′-methylenedioxyacetophenone, and 1,3-butanediol), and solvents (cyclohexane, propan-2-ol, benzene, and methanol) were procured through Sigma Aldrich, USA having a minimum purity of 99.0%.

### Sample collection and storage

Cigarettes and bidis from India and Myanmar were selected based on market availability and consumer popularity at the time of sampling, with an emphasis on including both local and well-known brands. Sampling was conducted randomly within the limits of regional availability. Samples were randomly selected from the cigarette sales points. Four cigarettes and nine Bidi brand samples were procured locally from wholesale and retail outlets in and around Bangalore, India. Seven cigarette brands sourced from Myanmar were obtained through the WHO South-East Asia Regional Office (SEARO), New Delhi, India. Due to the small sample size and random selection, our results may be considered preliminary or indicative. Reference cigarettes (3R4F, 1R5F, and 2R4F) were obtained from the University of Kentucky, USA. Cooperation Centre for Scientific Research Relative to Tobacco (CORESTA) Monitor (CM4 and CM6) reference cigarettes were received from CORESTA (Paris, France) (Table 3).

The samples were transported in air-tight sealed packs to the Tobacco Testing Facility, NIMHANS, Bengaluru. The labelled samples were stored in sealed packs within 10 days of receipt at –20 °C (Ultra-Low Deep Freezer, Vestfrost) in their original packaging. Before any analysis (e.g., TNCO, humectants, BaP, etc.), the samples were conditioned in a controlled environment as per the International Organization for Standardization (ISO) 3402, a critical pre-analytical step for reliable results^11,13^.

Cigarettes and bidis contain many chemicals, some occur naturally in tobacco, while others are formed during tobacco processing^11^. These products emit thousands of chemicals, including addictive substances (nicotine), toxicants (tar and CO), and carcinogens (BaP). A smoking machine is a valuable tool for researching human smoking patterns, even though it does not perfectly replicate every aspect of human smoking behavior. It allows for controlled, standardized smoke delivery, enabling researchers to isolate and study specific variables related to smoke exposure and its health effects. This machine simulates human smoking by controlling parameters such as puff volume, puff duration, puff frequency, and puff interval, allowing for the analysis of various factors^13,14^.

### Quantitative analytical measurements

All quantitative methods were performed under a strict quality assurance (QA) and quality control (QC) protocol in accordance with ISO 17025. For quantifying emission ingredients from both bidis and cigarettes, globally accepted procedures from the ISO, the CORESTA, the TobLabNet, and the Centers for Disease Control and Prevention (CDC) were followed (Table 1). All reported data were within the lowest and highest calibration range of the respective SOP. Values lower than the lowest calibration point were reported as “not detected”. All QCs were verified using a modified Westgard protocol^12^. Any data failing QC were excluded, and the measurements were repeated.

**Table 1:** Standard Operating Procedures applied for testing of cigarettes and bidis.

Parameter	Technique or SOP
Conditioning	ISO 3402
Smoking Machine (Cigarettes & Bidis)	ISO 3308 and ISO 17175
Carbon Monoxide (% by volume)	TobLabNet SOP 10
Total Particulate Matter	ISO 3308 and TobLabNet SOP 10
Water	CORESTA 15
Nicotine	TobLabNet SOP 10
Benzo[a]pyrene	TobLabNet SOP-5
Humectant	TobLabNet SOP 6
Flavours	CDC TL-Method 060
Quality Assurance/Quality Control	ISO 17025

### Smoking machine regime

The Cerulean SM450i, a 20-channel semi-automatic linear smoking machine designed to replicate human smoking behavior, was used for cigarette, bidis analysis. It complies with the ISO 3308^13^ standards, ensuring reliable and reproducible results (Table 1). The machine can handle a wide range of cigarettes and bidis without compromising efficiency or throughput. For bidi emission analysis, the ISO 17175^14^ protocol was applied. The smoking regime entailed puffing for two seconds at 30-s intervals. The Cerulean bidi holder (Part: 99301) and latex bidi sleeve (Part: KU0358) were used in conjunction with a handheld vacuum pump to smoke bidis (Table 2).

**Table 2:** Parameters of the smoking run using the Cerulean SM450i.

Parameters	Cigarettes	Bidis
Method	ISO 3308	ISO 17175
Puff volume (mL)	35	35
Puff duration (sec)	2	2
Puff interval (sec)	60	30
Puff frequency	1 puff every 60 s	1 puff every 30 s
Sample Size	20	10

### Tar, Nicotine, CO, and water

**Fig. 1:**
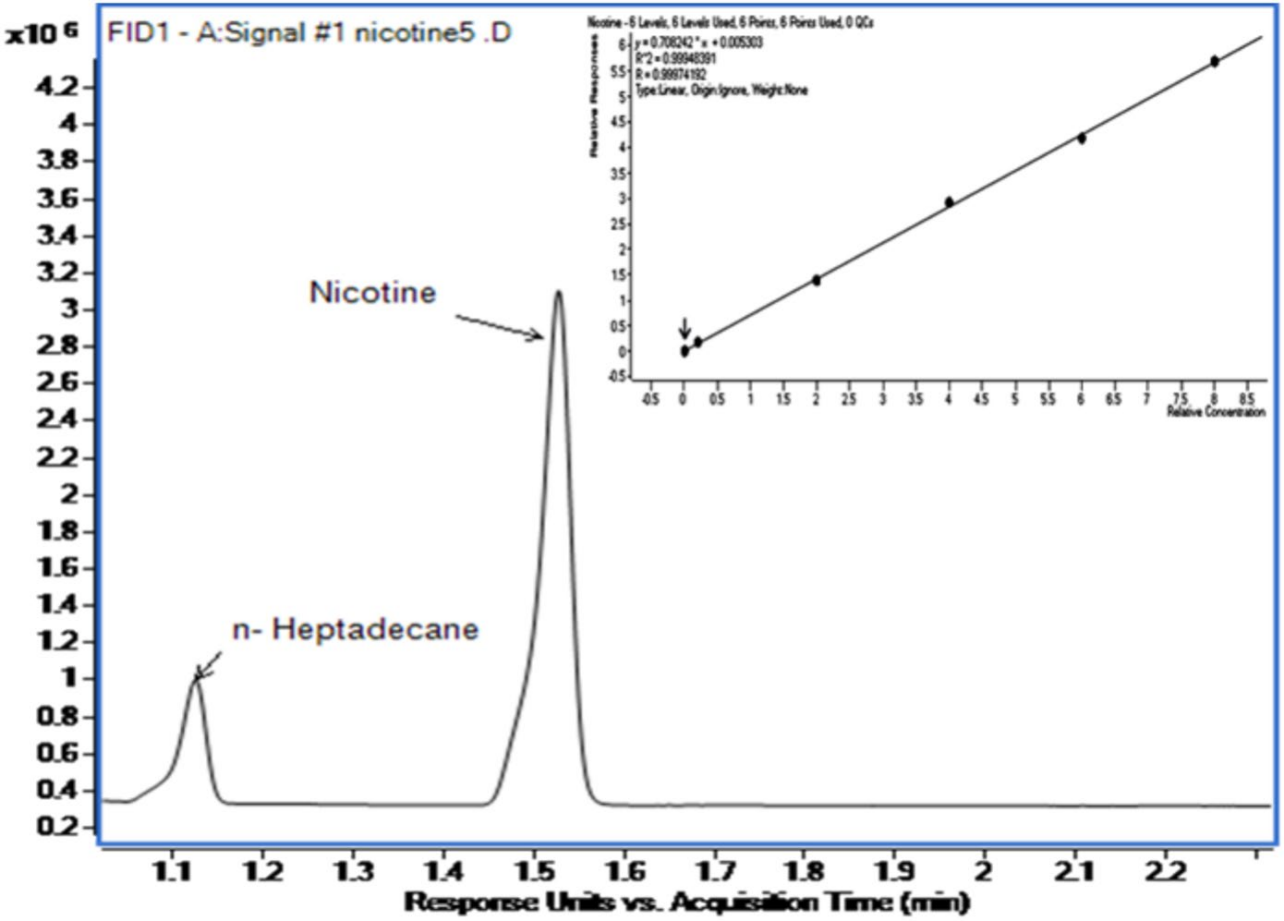
Chromatogram of nicotine with calibration curve.

The emissions were collected in bags, and the CO percentage by volume was measured using SOP 10 through the inbuilt non-dispersive infrared (NDIR) analyzer. Total Particulate Matter (TPM) was calculated as per ISO 3308 by weighing the holders before and after the smoking run^13^. The particulate matter collected on Cambridge Filter Pads (CFP, 44 mm) was extracted as per TobLabNet SOP 10^15^. Cigarette sampling was as per ISO 8243: 2013^16^, and water analysis was performed using CORESTA SOP 15^17^. Nicotine quantification was carried out on an Agilent 8890 Gas Chromatograph (GC) with a Flame Ionization Detector (FID). Chromatographic separation was achieved on a CP-WAX 51 column (CP7405) in isothermal mode at 170º C, as shown in Fig. 1.

### Humectants

**Fig. 2:**
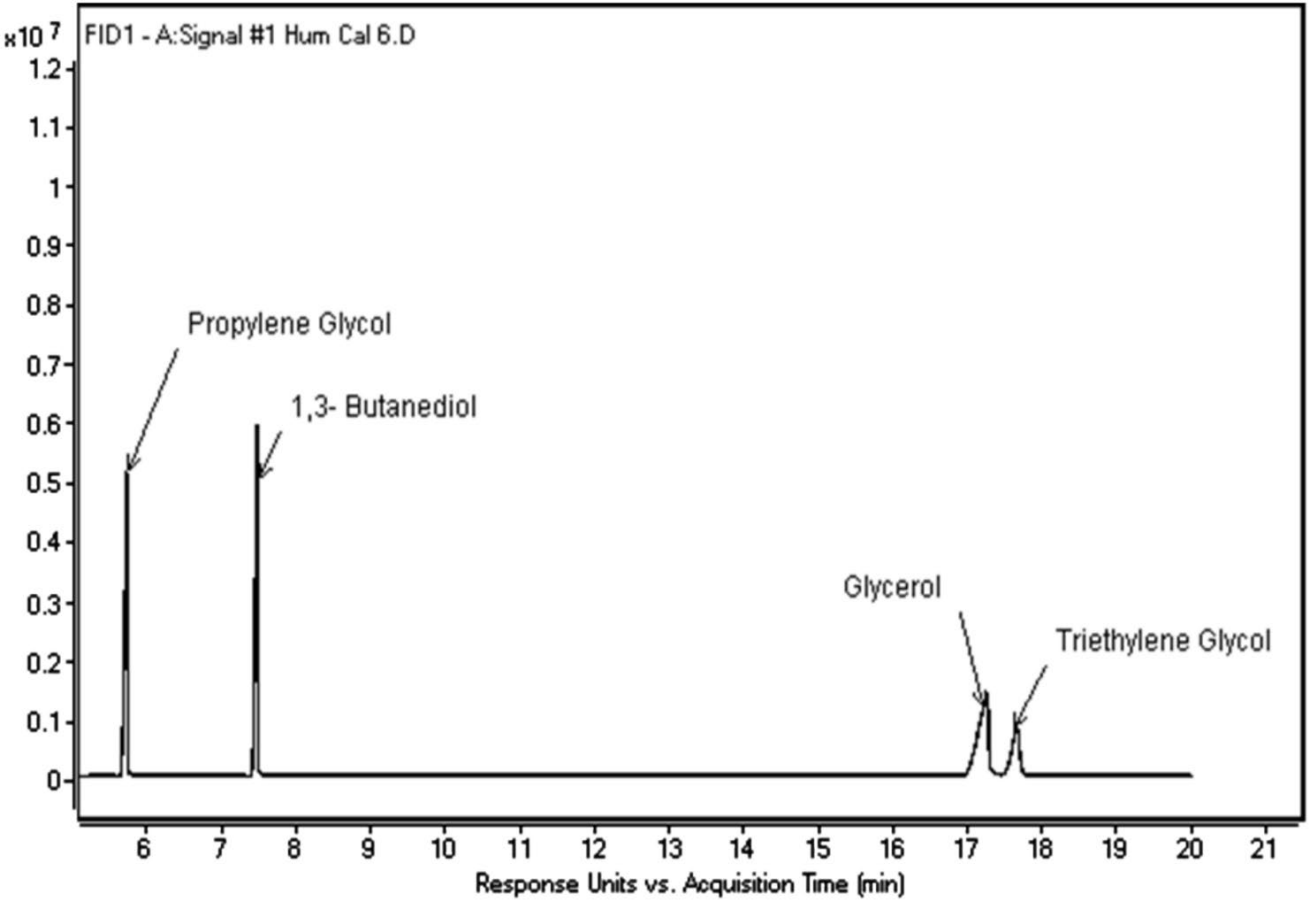
Chromatogram showing humectants.

Humectants (glycerol, propylene glycol, and triethylene glycol) were determined in cigarette tobacco fillers, and sampling was done as per ISO 8243^16^. The same SOP was followed for bidi analysis. For quantification, the TobLabNet SOP 6^18^ was applied using an Agilent 8890 GC equipped with a FID. The humectants were separated on a DB Wax (3–7032) fused silica column, as shown in Fig. 2.

### BaP

**Fig. 3:**
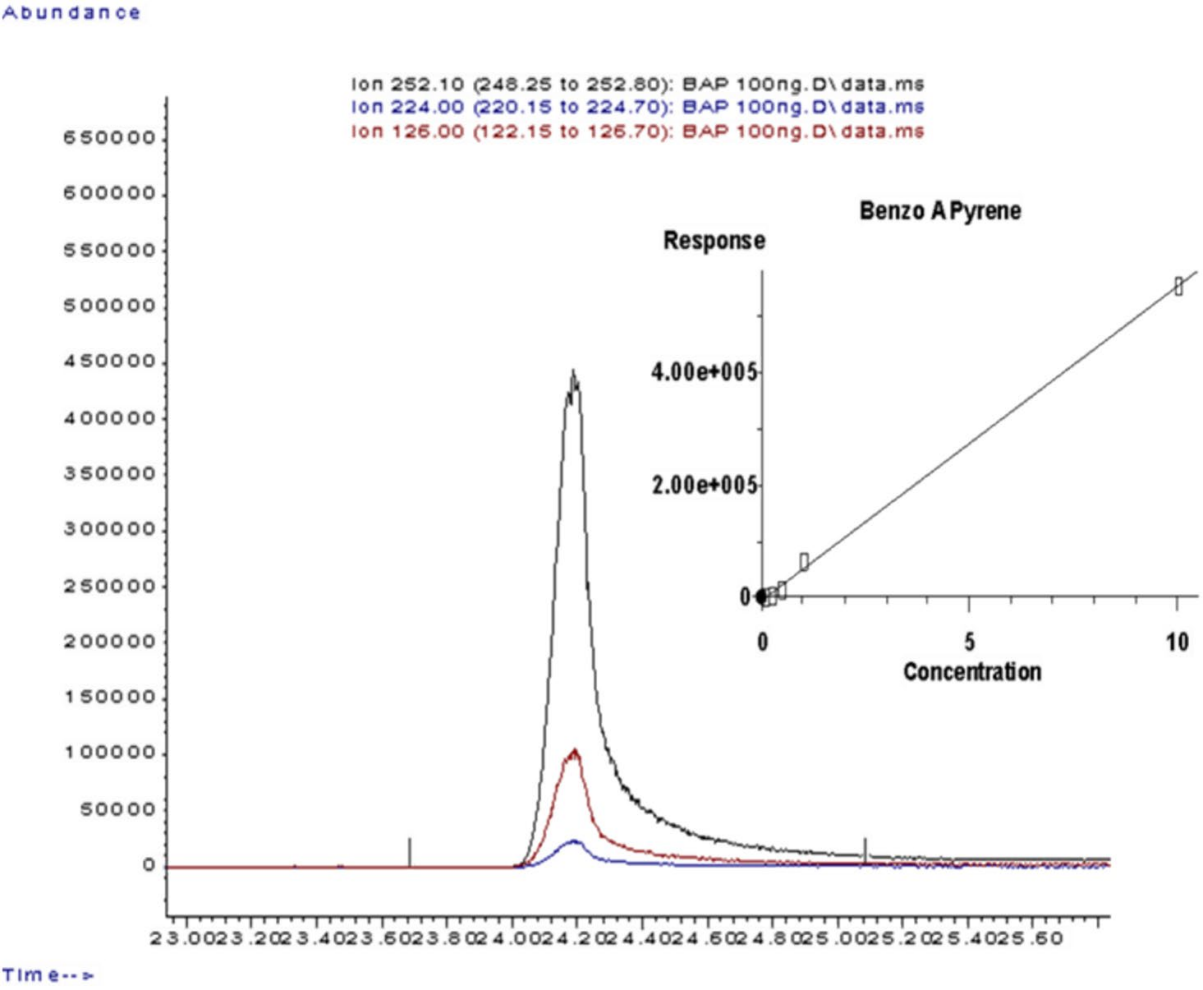
Chromatogram, m/z, and calibration curve of benzo[a]pyrene(BaP).

BaP was quantified using TobLabNet SOP 5^19^ on a 5975 Mass Selective Detector (MSD) coupled with an Agilent 7890 GC-MSD system. Chromatographic separation was achieved on a DB-5MS column (122-5532G). The mass spectra of ions produced by electron ionization were identified using the National Institute of Standards and Technology (NIST) library, as shown in Fig. 3.

### Statistical analysis

IBM SPSS Statistics for Windows, Version 23.0 (2015; IBM Corp., Armonk, NY, USA) was used to perform the statistical analysis. Due to the small sample size, non-parametric statistical methods were applied to compare cigarettes and bidis between the two countries. The Mann–Whitney U test was employed for the analysis, with *p* < 0.05 considered statistically significant.

## Results

Reference control samples (3R4F, 1R5F, 2R4F, CM4, and CM6) were studied in triplicate under comparable conditions, and the results are presented as means ( ~) in Table 3.

**Table 3:** Smoke constituents of reference cigarettes.

Reference cigarette	TPM (mg/cig)	Tar (mg/cig)	Nicotine (mg/cig)	Water (% filler)	Humectants (Glycerol & Propylene Glycerol)	BaP (ng/cig)
3R4F	~ 11.7	~ 9.4	~ 0.85	~ 13.2	Not detected	~ 2.18
1R5F	~ 6.8	~ 5.5	~ 0.42	~ 12.5	Not detected	~ 2.11
2R4F	~ 12.0	~ 10.2	~ 0.78	~ 13.0	Not detected	~ 2.09
CM4	~ 10.2	~ 8.4	~ 0.60	~ 12.5	Not tested	Not tested
CM6	~ 8.5	~ 6.8	~ 0.50	~ 12.4	Not tested	Not tested

### Indian cigarettes vs Myanmar cigarettes

#### Tar, Nicotine, CO, and Water

**Fig. 4:**
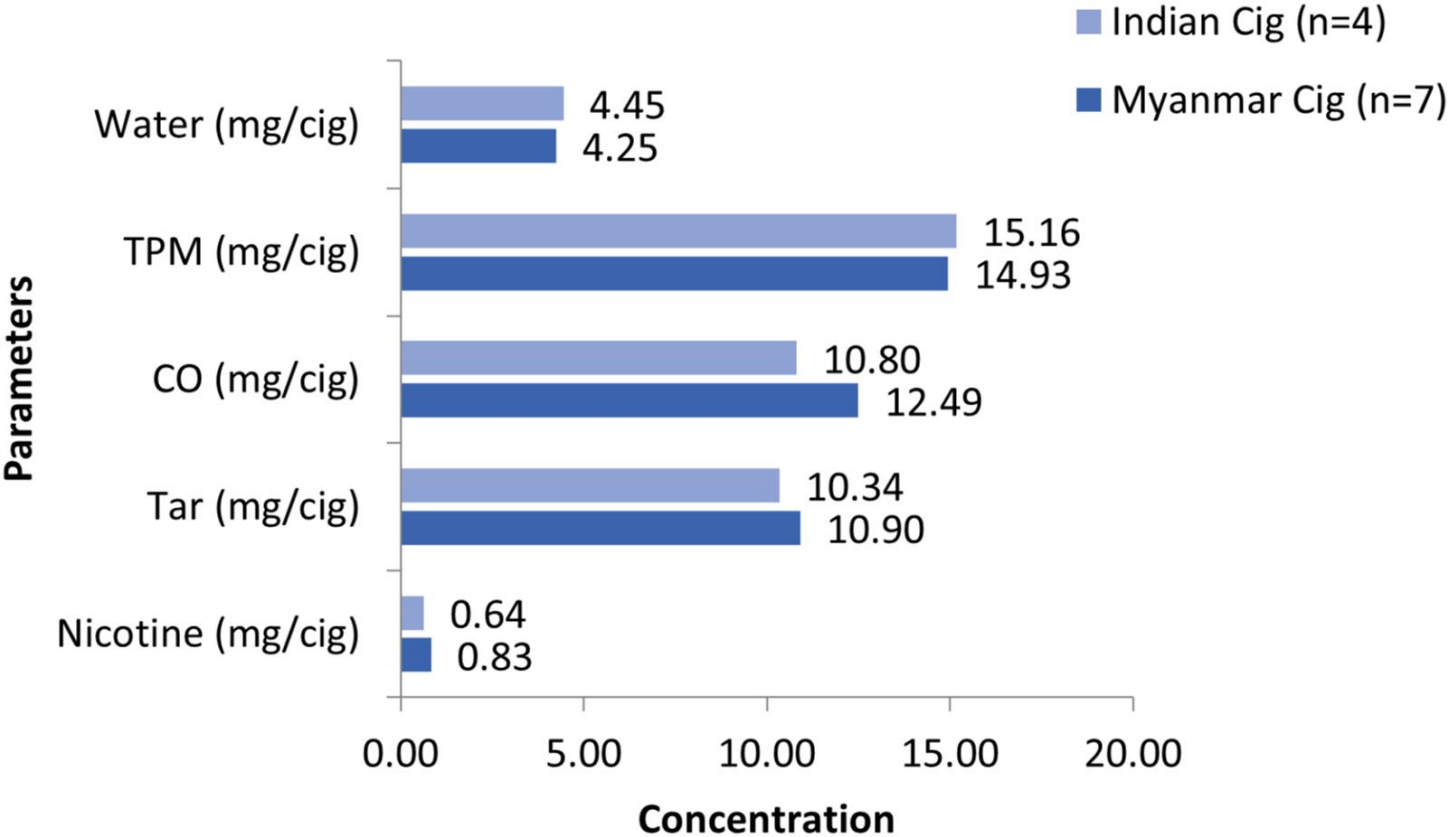
Medians of nicotine, water, carbon monoxide (CO), tar, and total particulate matter (TPM) in cigarettes.

The comparisons of nicotine, CO, water, TPM and tar between the cigarette samples received from Myanmar and India are summarized in Fig. 4. In Myanmar cigarettes, nicotine ranged from 0.59 to 1.08 mg/cigarette (median 0.83 mg/cigarette), while CO ranged from 7.77 to 18.96 mg/cigarette (median 12.49 mg/cigarette). In Indian cigarettes, nicotine ranged from 0.59 to 0.78 mg/cigarette (median 0.64 mg/cigarette), and CO ranged from 9.85 to 12.0 mg/cigarette (median 10.80 mg/cigarette). Although the nicotine and CO levels in Myanmar cigarettes were slightly higher than in Indian cigarettes, the differences were not statistically significant (*p* = 0.107 and *p* = 0.345, respectively). (see Supplementary file*cigarette_beedi data F.xlsx*).

The water content in Indian cigarettes ranged from 3.68 to 5.18 mg/cigarette (median 4.45 mg/cigarette), while for Myanmar cigarettes it ranged from 1.09 to 7.35 mg/cigarette (median 4.25 mg/cigarette); the difference was not statistically significant (*p* = 0.705). The tar and TPM in Indian products ranged from 8.49 to 12.22 mg/cigarette (median 10.34 mg/cigarette) and 13.41 to 18.04 mg/cigarette (median 15.16 mg/cigarette), respectively. For Myanmar products, tar ranged from 7.88–18.60 mg/cigarette (median 10.90 mg/cigarette) and TPM from 9.56 to 24.21 mg/cigarette (median 14.93 mg/cigarette); however, the differences were not statistically significant (*p* = 0.705) (Fig. 4; see Supplementary file *cigarette_beedi data F.xlsx*).

#### BaP

**Fig. 5:**
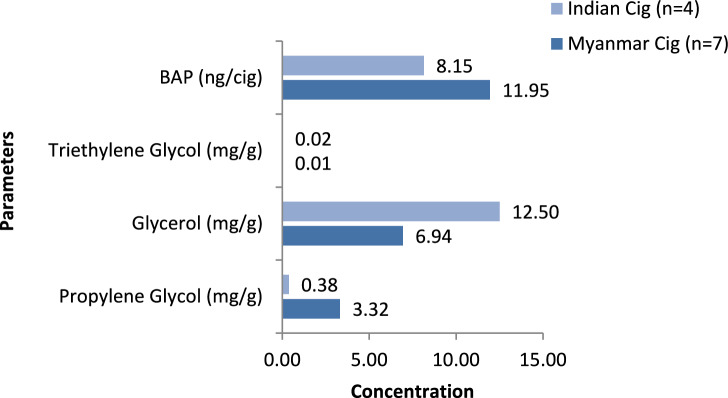
Medians of benzo[a]pyrene(BaP) and humectants in cigarettes.

As per our findings, BaP levels in Indian cigarettes ranged from 8.02 to 8.64 ng/cigarette (median 8.15 ng/cigarette), while Myanmar cigarettes showed higher BaP levels ranging from 9.72 to 14.90 ng/cigarette (median 11.95 ng/cigarette). The difference in BaP levels between Indian and Myanmar cigarettes was statistically significant (*p* = 0.008) (Fig. 5). Among the reference cigarettes, 3R4F, 1R5F, and 2R4F showed BaP levels ranging from 2.09 to 2.18 ng/cigarette (Table 3; see Supplementary file *cigarette_beedi data F.xlsx*).

#### Humectants

Humectants are added to cigarettes to control and maintain the moisture content of the tobacco filler. In Indian cigarettes, glycerol levels ranged from 1.99 to 14.11 mg/g (median 12.50 mg/g), propylene glycol from 0.02 to 0.75 mg/gm (median 0.38 mg/g), and triethylene glycol from 0.00 to 0.09 mg/g (median 0.02 mg/g). In Myanmar cigarettes, glycerol levels ranged from 5.46 to 17.36 mg/g (median 6.94 mg/g), propylene glycol from 0.42 to 3.86 mg/g (median 3.32 mg/g), and triethylene glycol from 0.00 to 0.07 mg/g (median 0.01 mg/g).

On comparing all three humectants, no statistically significant variation was observed in glycerol (*p* = 1.000) and triethylene glycol (*p* = 0.766); however, propylene glycol levels showed a statistically significant difference (*p* = 0.023) (Fig. 5). Humectants were not detected in the reference cigarettes (Table 3, see Supplementary file *cigarette_beedi data F.xlsx*).

#### Cigarettes vs bidis

**Fig. 6:**
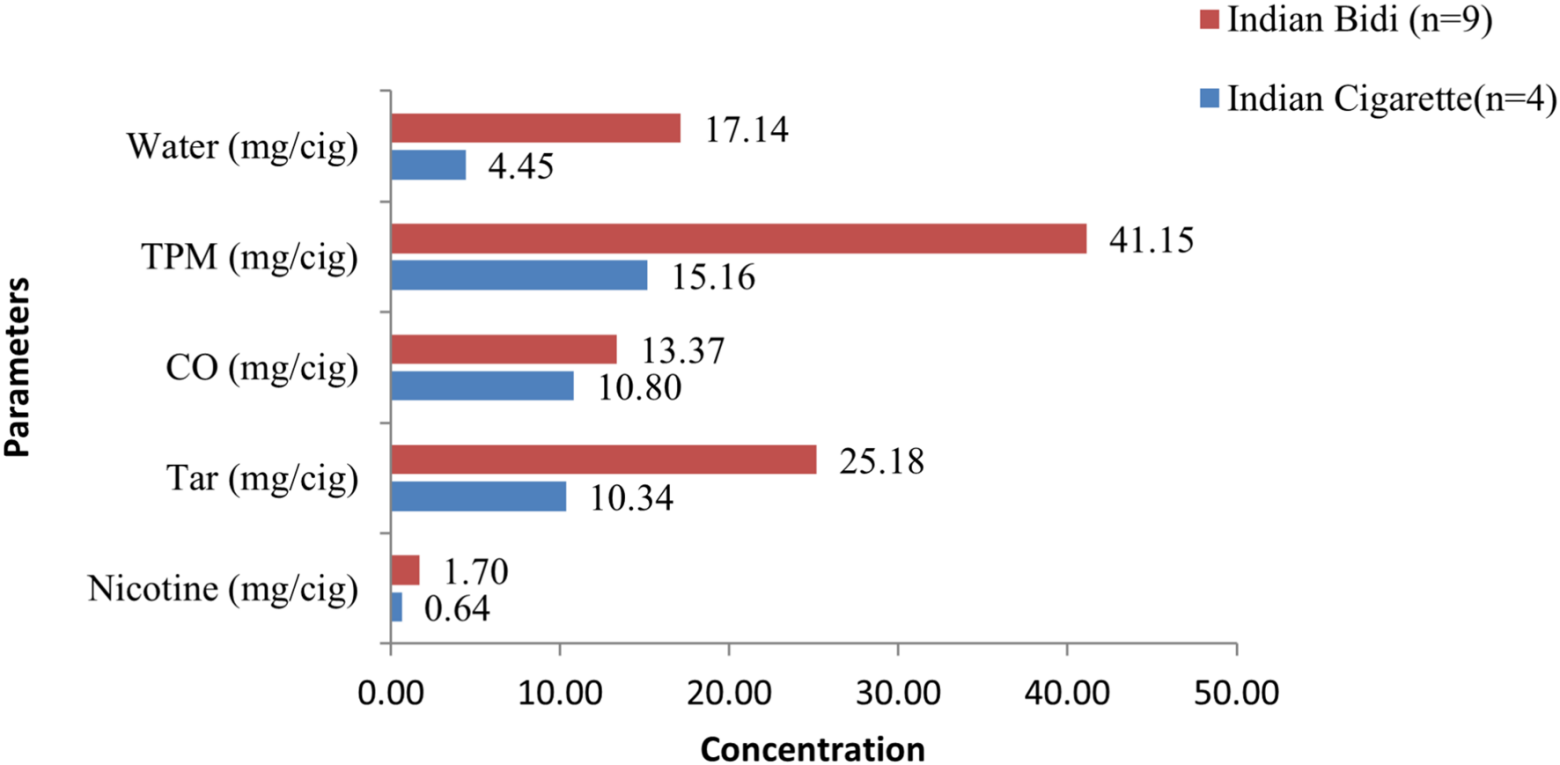
Medians of water, total particulate matter (TPM), carbon monoxide (CO), tar, and nicotine in Indian cigarettes and bidis.

Bidis had nicotine levels ranging from 0.69 to 1.91 mg/bidi (median 1.70 mg/bidi) and CO levels from 11.28 to 18.69 mg/bidi (median 13.37 mg/bidi) (Fig. 6). The water content of bidis varied from 1.32 to 28.40 mg/bidi (median 17.14 mg/bidi). Tar levels ranged from 1.84- 29.24 mg/bidi (median 25.18 mg/bidi), and TPM levels from 25.35- 48.09 mg/bidi (median 41.15 mg/bidi).

On comparing Indian cigarettes and bidis, nicotine and CO (*p* = 0.023), as well as water and TPM (*p* = 0.008), showed statistically significant differences. When comparing bidis and cigarettes from India and Myanmar, statistically significant differences were observed in nicotine (*p* = 0.041), water (*p* < 0.001), and TPM (*p* < 0.001) (see Supplementary file *cigarettes_beedi data F.xlsx*).

**Fig. 7:**
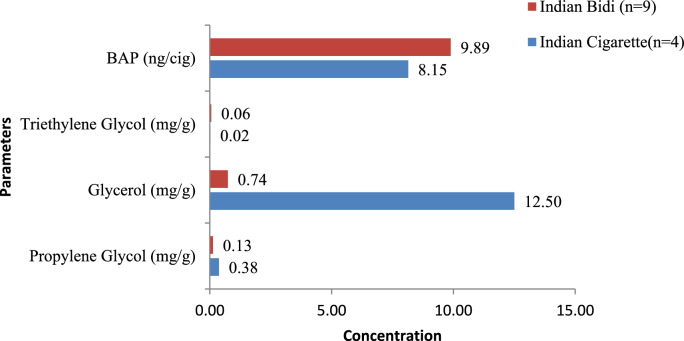
Medians of benzo[a]pyrene (BaP) and humectants in Indian cigarettes and bidis.

Bidis had glycerol levels ranging from 0.53 to 2.38 mg/g (median 0.74 mg/g), propylene glycol from 0.00 to 0.15 mg/g (median 0.13 mg/g), and triethylene glycol from 0.00 to 0.07 mg/g (median 0.06 mg/g). BaP levels in bidis ranged from 9.27 to 12.35 ng/bidi (median 9.89 ng/bidi). On comparing Indian bidis and cigarettes, glycerol and BaP levels were found to be statistically significant (*p* = 0.009 and p = 0.008, respectively), as shown in Fig. 7.

All cigarette and bidi emissions were tested for flavours (methyl salicylate, ethyl salicylate, eugenol, eucalyptol, diphenyl ether, cinnamaldehyde, menthol, coumarin, and camphor) using the CDC TL-Method 060^11^. None of these flavours were detected in the samples received from either country.

## Discussion

Cigarettes are expertly crafted, mass-produced tobacco products available worldwide. They are filled with processed tobacco and wrapped in paper. Bidis, in contrast, are small, hand-rolled tobacco products made of sun-dried and processed tobacco wrapped in *Tendu* or *Temburni* leaves and secured a with cotton thread^20^. India has the world’s second-largest number of adult smokers. Bidi is the most commonly smoked tobacco product, used by an estimated 71.8 million adults^1,2^.

Tobacco smoke is a complex mixture of over seven thousand volatile and semi-volatile compounds. Particulate matter (PM) generated from tobacco smoking can be coarse (≤ 10 µm), fine (≤ 2.5 µm), or ultrafine (< 0.1 µm)^20^. The particle size of PM determines both its ability to adsorb toxic organic compounds and the depth to which it can enter the respiratory system. Protonated nicotine is rapidly absorbed by the body. Other constituents of tobacco smoke include humectants, volatile aldehydes, nitrogen oxides, and polycyclic aromatic hydrocarbons. Hoffmann et al.^21^ identified N-nitrosonornicotine in unburned tobacco using gas chromatography–mass spectrometry (GC–MS) and reported it as a potential carcinogen at concentrations ranging from 0.12 to 3.7 µg/cigarette. Many harmful and potentially carcinogenic substances are present in mainstream smoke. When inhaled, high levels of carbon monoxide, especially from bidis, enter the bloodstream and bind with hemoglobin to form carboxyhemoglobin. This results in tissue ischemia, a major contributor to cardiovascular diseases common among bidi smokers^20,22^.

The Government of India signed the WHO FCTC in 2004^9^ and enacted the Cigarettes and Other Tobacco Products Act (COTPA) in 2003. However, COTPA does not impose any specific limits on tobacco product emissions or nicotine content. In its advisory note on the Global Nicotine Reduction Strategy, the TobReg^8^ stated that the risk of dependence on cigarettes can be reduced by lowering their nicotine content to very low levels. Accordingly, the nicotine content should be maintained as low as is technically feasible.

According to WHO’s global review of national laws^2,4,10^ on maximum permissible emission levels of tar, nicotine, and CO, as of 31 December 2022, eighty-three countries had established maximum permissible nicotine levels per cigarette, with sixty of these (72%) allowing up to 1 mg of nicotine per cigarette. Furthermore, sixty-seven countries permit 10 mg of tar per cigarette, while eighty-five countries have set maximum permissible tar levels per cigarette. Additionally, fifty-seven countries have set a CO limit of 10 mg per cigarette. Within the WHO SEAR, only Myanmar expressly forbids the display of emission levels, whereas Timor-Leste has established limits of 10 mg for tar, 1 mg for nicotine, and 10 mg for CO per cigarette^10^.

The cigarettes tested displayed nicotine concentrations between 0.59 and 1.08 mg per cigarette, with a median value of 0.77 mg per cigarette; all measured levels were within the regulatory threshold of 1.0 mg per unit^22^. For bidis, nicotine levels ranged from 0.69 to 1.91 mg per bidi (median 1.70 mg per bidi), which were well above 1.0 mg per unit^20,22^. Cigarette samples received from Myanmar were found to contain carbon monoxide (CO) levels exceeding 10 mg per cigarette. For bidis, CO levels ranged from 11.28 to 18.69 mg per bidi (median 13.37 mg per bidi). Under standard smoking machine conditions, bidis exhibited higher nicotine delivery, suggesting greater exposure potential. Research has shown that the non-porous nature and higher moisture content of *Tendu* leaf wrappers in bidis lead to higher levels of CO and tar in their smoke compared with regular cigarettes^20^. The presence of harmful and carcinogenic chemicals in mainstream bidi smoke can pose serious risks to human health^12^.

The tar levels in Myanmar cigarettes ranged from 7.88 to 18.60 mg per cigarette (median 10.90 mg per cigarette), exceeding the highest permissible limit of 10.0 mg per cigarette set by several countries. Indian bidis exhibited tar levels ranging from 1.84 to 29.24 mg per bidi (median 25.18 mg per bidi), demonstrating substantially higher values compared to typical manufactured cigarette standards. Humectants, propylene glycol, and glycerol were present in all samples. Among Indian cigarettes, propylene glycol levels did not exceed 10.0 mg/g, while glycerol levels were 10 mg/g or higher, with the exception of one brand. Triethylene glycol was detected only in two samples, ranging from 0.04 to 0.09 mg/g.

Yan Xizheng et al. tested twenty-seven popular cigarette brands in the United States of America (USA) for humectants. Only four brands showed no detectable amounts of humectants, while the remaining brands contained glycerol or 1,2-propylene glycol with concentrations ranging from 1.66 to 3.57% for glycerol and 0.23 to 1.35% for 1,2-propylene glycol. In general, all manufacturers used higher amounts of glycerol than 1,2-propylene glycol as a humectant, which may act as a precursor for the formation of harmful carbonyl compounds^23^.

BaP is a marker of carcinogenic activity of polycyclic aromatic hydrocarbons (PAHs)^24^. PAHs are present in cigarette smoke emissions in small quantities, typically < 10 ng per cigarette. BaP levels in Indian cigarettes were ≤ 10 ng per cigarette. In the Myanmar samples, BaP ranged from 9.72 to 14.90 ng per cigarette (median 11.95 ng per cigarette), which may be harmful depending on frequency and duration of use. Pakhale S.S. et al. analyzed mainstream smoke from popular Indian tobacco products using a standard smoking machine and reported BaP levels ranging from 85 to 114 ng per cigarette, while for bidis, levels ranged from 108 to 144 ng per bidi^25^. The lower BaP levels observed in the current study may be attributed to improved analytical techniques, differences in smoking machine regimens, and variations in product design or combustion conditions. Despite containing less tobacco, bidis deliver significantly higher levels of harmful and carcinogenic chemicals, including nicotine, CO, and PAHs^25^. Analysis of mainstream smoke in cigarettes from Nigeria^20^ revealed BaP levels between 0 and 22.7 ng per cigarette. The most prevalent PAH detected across all tested products was naphthalene, which ranged from 210.7 to 460.34 ng per cigarette^24^. Unlike conventional cigarettes, for which more than 6500 tobacco-specific chemicals have been identified and studied in detail^25^, research on bidi smoke remains limited. Therefore, more studies are required to characterize the complete chemical profile of bidis and assess their health impact.

## Conclusion

Cigarettes and bidis pose a significant public health challenge. Their wide availability and continued appeal may overshadow the serious health risks associated with these products. Quantitative analysis of emissions in mainstream smoke is necessary to assess the harmful effects of smoking. In the present study, we quantitatively analyzed nicotine, CO, water, tar, TPM, and BaP in mainstream smoke from randomly selected cigarette (India and Myanmar) and bidi (India) samples. These samples were analyzed using globally accepted SOPs developed by the ISO, CORESTA, and TobLabNet. Statistically significant differences were observed in BaP (*p* = 0.008) and propylene glycol (*p* = 0.023) levels between Indian and Myanmar cigarettes. When comparing Indian cigarettes with bidis, nicotine and CO (*p* = 0.023), water and BaP (*p* = 0.008), and glycerol (*p* = 0.009) showed statistically significant variation.

The contents and emissions were measured using globally approved protocols. The results of this study can be utilized to inform policy formulation, educate the public about the dangers of smoking, and potentially incentivize tobacco companies to produce less harmful products by adhering to established emission standards for toxic constituents.

### Limitation

Analysis of tobacco-specific nitrosamines could not be performed due to non-availability of reference standards. Also, the overall findings of this study should be considered preliminary or indicative, given the limited sample size.

## Supplementary Information

Supplementary Information.

## Data Availability

The datasets used and/or analysed during the current study are available from the corresponding author upon reasonable request.

## Abbreviations

BaP: Benzo[a]pyrene
CAM: Centre for addiction medicine
CDC: Centers for disease control and prevention
CFP: Cambridge filter pad
CO: Carbon monoxide
CORESTA: Cooperation centre for scientific research relative to tobacco
COTPA: Cigarettes and other tobacco products act
FCTC: Framework convention on tobacco control
FID: Flame ionization detector
GC: Gas chromatograph
GC–MS: Gas chromatography-mass spectrometry
ISO: International organization for standardization
MSD: Mass selective detector
NDIR: Non- dispersive infrared analyzer
NIMHANS: National institute of mental health and neurosciences
PAH: Polycyclic aromatic hydrocarbon
PM: Particulate matter
QA: Quality assurance
QC: Quality control
SEAR: South-east asia region
SEARO: South-east asia regional office
SOP: Standard operating procedure
TFI: Tobacco free initiative
TNCO: Tar, nicotine, and carbon monoxide
TobLabNet: Tobacco laboratory network
TobReg: WHO study group on tobacco product regulation
TPM: Total particulate matter
WHO: World health organization

## References

[1] Kaur, J., Rinkoo, A. V. & Richardson, S. Update on numbers of tobacco-attributable deaths by country in the South-East Asia region: implications for policy. *Tob. Control.* **34**(4), 544–547. 10.1136/tc-2024-058599 (2025). 38851291 10.1136/tc-2024-058599

[2] WHO report on the global tobacco epidemic, 2017: monitoring tobacco use and prevention policies. Geneva: World Health Organization; 2017. Licence: CC BY-NC-SA 3.0 IGO (Global Burden of Disease [database. Washington, DC: Institute of Health Metrics; 2019. IHME, Accessed (17 Jul 2023).

[3] Chen, D.T.-H. et al. A longitudinal study of transitions between smoking and smokeless tobacco use from the ITC Bangladesh Surveys: implications for tobacco control in the Southeast Asia region. *Lancet Reg. Health Southeast Asia* **14**, 100185 (2023). 37492418 10.1016/j.lansea.2023.100185 PMC10363488

[4] WHO global report on trends in prevalence of tobacco use 2000–2025, fourth edition. ISBN 978-92-4-003932-2

[5] John, R. M., Sinha, P., Munish, V. G. & Tullu, F. T. Economic Costs of Diseases and Deaths Attributable to Tobacco Use in India, 2017–2018. *Nicotine Tob. Res.* **23**(2), 294–301. 10.1093/ntr/ntaa154 (2021). 32805055 10.1093/ntr/ntaa154

[6] Murray, J. B. Nicotine as a psychoactive drug. *J. Psychol.* **125**(1), 5–25. 10.1080/00223980.1991.10543265 (1991). 2033559 10.1080/00223980.1991.10543265

[7] Action on Smoking and Health. What’s in a Cigarette. (2022).https://Ash.Org.Uk/Resources/View/Whats-in-a-Cigarette

[8] Tobacco Free Initative. Global network. WHO Study Group on Tobacco Product Regulation (TobReg). Available at: http://www.who.int/tobacco/global_interaction/ tobreg/en/. Accessed (13 Jan 2015.

[9] World Health Organization (WHO) Framework Convention on Tobacco Control. Tobacco, Data and Statistics, Adults. 2009. Available at: http://www.euro.who.int/en/health-topics/disease-prevention/tobacco/data-andstatistics/who-is-smoking/adults/. Accessed (13 Jan 2015).

[10] Reddy, K. S. & Gupta, P. C. Report on Tobacco Control in India. In *Ministry of Health & Family Welfare, Government of India* (ed. Gupta, P. C.) (WHO, 2004).

[11] Sharma, P. et al. Physical and chemical characterization of smokeless tobacco products in India. *Sci. Rep.* **13**, 8901. 10.1038/s41598-023-35455-3 (2023). 37264008 10.1038/s41598-023-35455-3 PMC10235085

[12] Westgard, J. O., Barry, P. L., Hunt, M. R. & Groth, T. A multi-rule Shewhart chart for quality control in clinical chemistry. *Clin. Chem.* **27**, 493–501 (1981). 7471403

[13] International Organization for Standardization. (2012). ISO 3308:2012 - Routine analytical cigarette-smoking machine — Definitions and standard conditions. Geneva, Switzerland: ISO.

[14] International Organization for Standardization. (2006). ISO 17175:2006 – Tobacco and tobacco products — Guidance for correlative studies of analytical results and health-related data. International Organization for Standardization. https://www.iso.org/standard/31280.html.

[15] World Health Organization Tobacco Laboratory Network (TobLabNet). Standard operating procedure for determination of nicotine and carbon monoxide in mainstream cigarette smoke under intense smoking conditions (WHO TobLabNet SOP 10, 2016.

[16] International Organization for Standardization. (2013). ISO 8243:2013 Cigarettes — Sampling (5th ed.). https://www.iso.org/standard/60154.html.

[17] CORESTA Recommended Method No. 15. Cigarettes – Determination of Water in Smoke Condensates by Karl Fischer Method. March 1990. CORESTA (Cooperation Centre for Scientific Research Relative to Tobacco).

[18] World Health Organization Tobacco Laboratory Network (TobLabNet). Standard Operating Procedure for Determination of Humectants in Cigarette Tobacco Filler (SOP 6). Geneva: WHO, (2016).

[19] World Health Organization Tobacco Laboratory Network (TobLabNet). Standard Operating Procedure No. 5: Determination of BAP in mainstream cigarette smoke under ISO and intense smoking conditions. Geneva: WHO, (3 Jun 2015).

[20] Oladipupo, O. A., Dutta, D. & Chong, N. S. Analysis of chemical constituents in mainstream bidi smoke. *BMC Chem.* **13**, 93 (2019). 31384840 10.1186/s13065-019-0614-7 PMC6661734

[21] Hoffmann, D., Hecht, S. S., Ornaf, R. M. & Wynder, E. L. N’-nitrosonornicotine in tobacco. *Science* **186**(4160), 265–267. 10.1126/science.186.4160.265 (1974). 4414773 10.1126/science.186.4160.265

[22] Kienhuis, A., Klerx, W. & Talhout, R. Regulation of Emissions of Tobacco Products other than Cigarettes. *Tobacco Regul. Sci.* **1**, 142–153 (2015).

[23] Xizheng, Y., Valentín-Blasini, L., Watson, C. & Cardenas, R. B. Determination of Humectants in Tobacco Filler by High Performance Chromatography/Single Quadrupole Mass Spectrometry. *Beiträge zur Tabakforschung Inter. Contr. Tobacco Res.* **28**, 170–178 (2018). 10.2478/cttr-2018-0016 PMC11160111 38854422

[24] Adesina, O. A., Olowolafe, T. I. & Igbafe, A. Levels of polycyclic aromatic hydrocarbon from mainstream smoke of tobacco products and its risks assessment. *J. Hazardous Materials Adv.* **5**, 100053 (2022).

[25] Pakhale, S. S., Jayant, K. & Bhide, S. V. Chemical analysis of smoke of Indian cigarettes, bidis and other indigenous forms of smoking–levels of steam-volatile phenol, hydrogen cyanide and benzo(a)pyrene. *Indian J. Chest Dis. Allied. Sci.* **132**(2), 75–81 (1990). 1964673

